# Rejection in Bargaining Situations: An Event-Related Potential Study in Adolescents and Adults

**DOI:** 10.1371/journal.pone.0139953

**Published:** 2015-10-07

**Authors:** Kiki Zanolie, David de Cremer, Berna Güroğlu, Eveline A. Crone

**Affiliations:** 1 Institute of Psychology, Leiden University, Leiden, the Netherlands; 2 Leiden Institute for Brain and Cognition (LIBC), Leiden University, Leiden, the Netherlands; 3 Judge Business School, Cambridge University, Cambridge, United Kingdom; Radboud University Nijmegen, NETHERLANDS

## Abstract

The neural correlates of rejection in bargaining situations when proposing a fair or unfair offer are not yet well understood. We measured neural responses to rejection and acceptance of monetary offers with event-related potentials (ERPs) in mid-adolescents (14–17 years) and early adults (19–24 years). Participants played multiple rounds of the Ultimatum Game as proposers, dividing coins between themselves and a second player (responder) by making a choice between an unfair distribution (7 coins for proposer and 3 for responder; 7/3) and one of two alternatives: a fair distribution (5/5) or a hyperfair distribution (3/7). Participants mostly made fair offers (5/5) when the alternative was unfair (7/3), but made mostly unfair offers (7/3) when the alternative was hyperfair (3/7). When participants’ fair offers (5/5; alternative was 7/3) were rejected this was associated with a larger Medial Frontal Negativity (MFN) compared to acceptance of fair offers *and* rejection of unfair offers (7/3; alternative was 3/7). Also, the MFN was smaller after acceptance of unfair offers (7/3) compared to rejection. These neural responses did not differ between adults and mid-adolescents, suggesting that the MFN reacts as a neural alarm system to social prediction errors which is already prevalent during adolescence.

## Introduction

A bargaining situation involves the distribution of valued goods between two parties where the proposed distribution has consequences for both self and other. Because of this structure, individuals in bargaining games form expectations about whether proposed distributions will be accepted by others or not [1, 2], which influences their subsequent behavior (i.e. self-focused behavior versus reciprocity). Bargaining plays an important role during emerging adulthood, the life phase between approximately 15 and 25 years, when individuals develop complex social relations, gain independence from their parents and develop mature social [3, 4]. Even though a basic sense of fairness in bargaining is already observed in young children [5, 6], the understanding of intentionality in social interactions develops gradually over the course of adolescence and early adulthood [6, 7]. As such, a crucial question concerns how individuals develop sensitivity to rejection and acceptance in this life stage, which is thought to be an inflection point for developing positive or negative future relations [3].

A task often used to study bargaining is the Ultimatum Game [8]. In this economic exchange game a proposer receives a given sum of money, the stake, and is asked to share the stake by offering a certain amount of the stake to the responder. If the responder accepts the offer, both players keep the amount allocated by the proposer. However, if the responder rejects the offer, both players go empty-handed. Based on economic rationality hypothesis [9], one would expect that responders accept all offers higher than nothing to maximize their personal gain. However, responders reject most offers lower than 30% of the share and exhibit a strong preference for fair offers hovering around a 50–50 split e.g. [10, 11,12, 2]. This task provides a valid context for examining the experience of bargaining rejection (e.g. [13, 14], see [15] for a review), but to date almost nothing is known about how proposers experience the rejection of offers.

Prior research examined the neural responses in Ultimatum Game bargaining from the perspective of the responder [16], by focusing on modulations of a negative event-related scalp potential (ERP), referred to as the medial frontal negativity (MFN). The MFN reflects a broad class of ERP responses referring to the medial frontal scalp distribution they share. The MFN is observed approximately 250–350 msec following the presentation of performance feedback and has previously been associated with, for example, negative prediction errors, losses in gambling tasks, and negative social outcomes (e.g. [17, 18], see [19] for a review) and as such provides a sensitive index of general performance monitoring [18]. Receiving unfair compared to fair offers in a bargaining game is associated with an increased negative MFN [16,17, 20, 21, 22,23]. The level of unfairness modulates the MFN, that is, the MFN is more pronounced for highly unfair offers than moderately unfair offers [16, 22, 24]. Individual differences analysis showed that especially participants with a high concern for fairness showed higher MFN responses to receiving unfair offers [17]. Here we test the hypothesis that rejection of a fair offer made by the proposer leads to a more negative MFN in the proposers, similar to the responders’ experience of receiving an unfair offer [13, 14].

In the current study, the participants between 15 years and 23 years of age played the role of the proposer in an experimental version of the Ultimatum Game [25] and made offers to another individual whom they met prior to the study. Participants played multiple rounds with the same player to build up social expectations [20, 21,22]. Participants could choose between an unfair offer (7/3, beneficial for the proposer but not for the responder) and an alternative offer that could either be a fair offer (5/5, an equal split of money) or a hyperfair offer (3/7, beneficial for the responder but not for the proposer). After every offer, the participant (proposer) received feedback to indicate whether the offer was accepted or rejected by the responder. We expected that when an unfair distribution (7/3) was contrasted with the fair distribution (5/5) participants would mainly make fair offers [6, 26], but when the unfair distribution (7/3) was contrasted with the hyperfair distribution (3/7) participants would mainly make unfair offers [13, 25]. We hypothesized that a larger MFN would be observed when offers were rejected compared to accepted by the responder, but more so when the proposed offer was fair (5/5), reflecting a violation of expectations that fair offers would be accepted [21, 27]. Given that intentionality understanding changes during adolescence [6, 7], we tested whether these neural responses would differ depending on whether participants were in adolescence (15 years) or in early adulthood (23 years).

## Methods

### Participants

Participants were 41 healthy undergraduate students of Leiden University and adolescents of local schools. Four adults and two adolescents were excluded from the analyses because they did not believe the cover story, showed no variation in their behavioral responses, or did not have enough ERP segments in one of the critical conditions. This resulted in a final sample of 18 adults (age: range = 19–24, *M* = 20.61, *SD* = 1.15; 8 female; 16 right-handed) and 16 adolescents (age: range = 14–17, *M* = 15.19, *SD* = 0.91; 11 female; 15 right-handed; handedness scale [28]) who were included in the analyses. All participants gave written informed consent for participation in the study; written informed consent was also obtained from the parents of the adolescents. None of the participants reported any psychiatric or neurological conditions or impairments and all participants had normal or corrected-to-normal vision. Participants received financial compensation for their participation. The study was approved by the Leiden University ethics committee.

### Design and procedure

Participants played a modified version of the Ultimatum Game (UG). They were instructed that they would play a game with another person of same age and gender. In reality, they played against the computer. We informed the participants that they would be the proposer during the entire game and that the other player would be the responder. To increase credibility of playing against another person, during preparation of the EEG equipment, a confederate walked into the room to ask how long preparations would take before the experiment could start, upon which participants were told that this was the experimenter responsible for instructing the other player. Additionally, fake calls were made between the two labs before starting the experiment and after each break.

**Fig 1:**
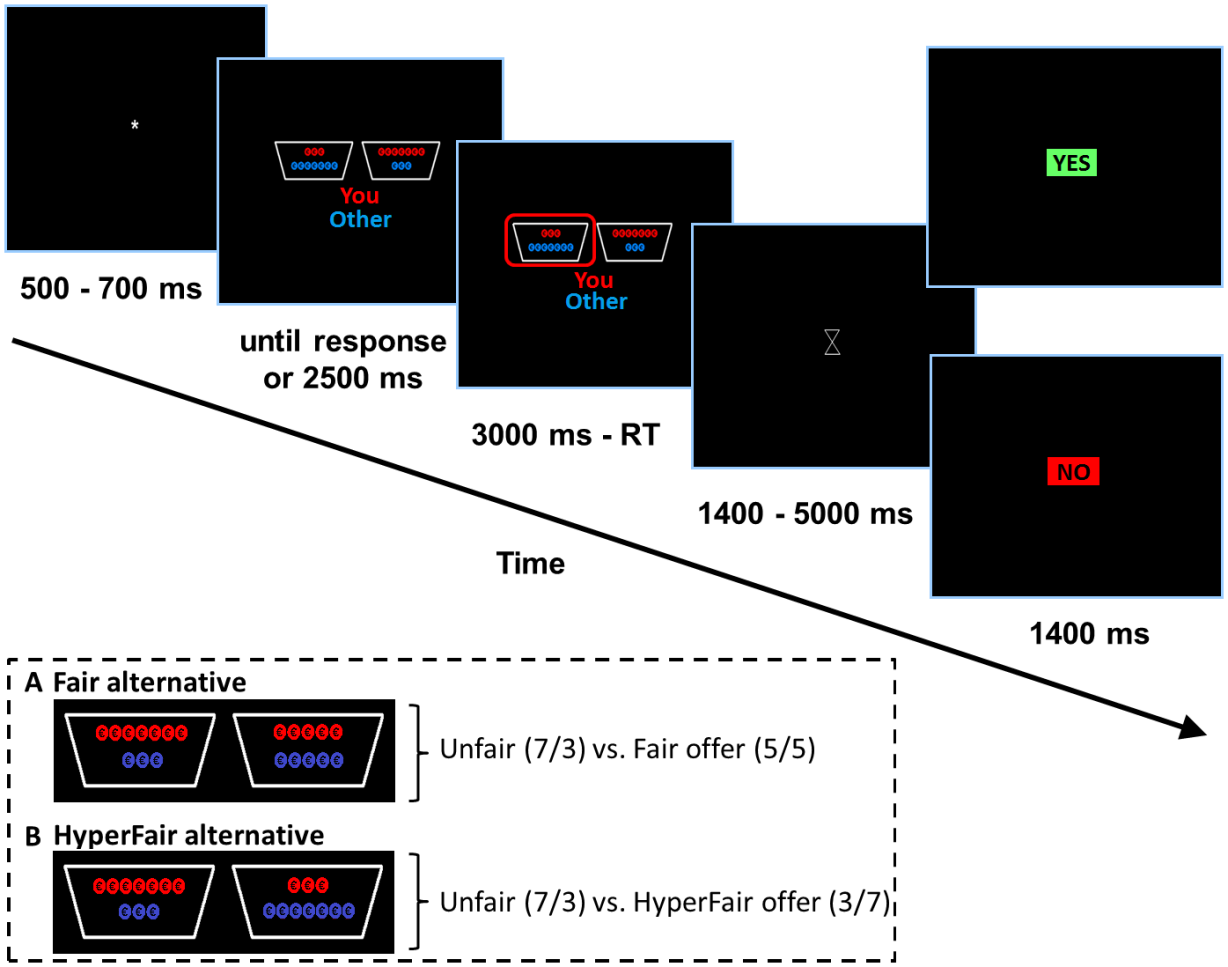
Task display. The task starts with a jittered fixation (500–700 ms). Then two distributions containing red coins (for the proposer) and blue coins (for the responder) are displayed (here the hyperfair-alternative condition depicted). This screen is displayed until a response is registered (maximum of 2500 ms). Upon response, the decision of the participant is highlighted by a red outline (2500 ms minus the reaction time). Following a jittered waiting screen (1400–5000 ms) the participant received feedback whether the offer was accepted (green box with YES) or rejected (red box with NO) for the duration of 1400ms. In the dotted panel the two conditions (A. fair-alternative; B. hyperfair-alternative) are displayed.

In the current version of the Ultimatum Game two sets of dichotomous distributions for sharing the stake between the proposer and responder were presented. For a visual display of these distributions see Fig 1. In both sets the unfair distribution of seven coins for the proposer and three coins for the responder (hereafter 7/3 offer) was pitted against an alternative offer: (i) the fair-alternative (5/5 offer), or (ii) the hyperfair-alternative (3/7 offer). The participant was informed that the other player saw the same screen as the proposer and, thus, was aware of which two distributions the participant could choose from. Participants were instructed that the coins won during the game would be converted into real money and paid out at the end of the study.

The acceptance and rejection rate of the offers was manipulated such that in the fair-alternative condition, approximately 60% of the fair offers and 40% of the unfair offers was accepted. In the hyperfair-alternative condition, approximately 60% of the hyperfair offers and 40% of the unfair offers was accepted. Acceptance or rejection of the responder followed an algorithm allowing the responses to be random, but falling within a 60–40% division.

Each trial started with a jittered 500–700 ms fixation cross. After the fixation, two alternative distributions were presented (symmetrically positioned compared to fixation). The visual angel between fixation and the end of a distribution was 5.5°. The visual angel of the total height of the distribution was 4.0°. Participants had a maximum of 2500 ms in which they could choose which of the two distributions to offer. If the participant did not make an offer within 2500 ms a feedback screen with “Too late” was presented for 1200 ms. The position of the unfair offer was counterbalanced in each condition. Participants could make an offer with a button press with their left middle or index finger. Upon response the chosen distribution was outlined in red for 800 ms plus the remaining time of the offer presentation (2500 ms—reaction time). Next, a waiting screen with a fixation cross was presented for 1400–5000 ms. During this waiting screen the fictive responder made a choice to accept or reject the offer. After this waiting screen, the feedback of the responder (i.e., acceptance or rejection of the offer) was presented for 1400 ms. This was done by the words”YES” or “NO” in the center of the screen to indicate acceptance or rejection of the offer, respectively. Subsequently, a new trial started.

Participants played eight practice trials. The experimental task itself consisted of four blocks. Each block consisted of 52 trials in which the fair alternative (5/5 vs 7/3) and hyperfair alternative (3/7 vs 7/3) conditions were presented 26 times each, in a random order, resulting in 208 trials in total. The total duration of the experiment, including self-paced breaks between blocks during which the participant remained seated, was approximately 35 minutes. Participants completed a funnel interview at the end of the study to test whether they believed the cover story and were subsequently debriefed.

### EEG data acquisition

The EEG signals were recorded through an Active-Two amplifier system (Biosemi, Amsterdam, The Netherlands) from 21 scalp electrodes according to the 10–20 system (Fz, F1/2, FCz, F3/4, FC1/2, FC3/4, Cz, C1/2, C3/4, CPz, CP1/2, Pz, P1/3). The 21 Ag/AgCl electrodes were mounted in an elastic cap. Six additional electrodes were attached to the left and right mastoids, two outer canthi of the eyes (HEOG), and infraoribital, and supraorbital regions of the left eye (VEOG). Two additional scalp electrodes were used to serve as reference and ground electrodes. Online signals were recorded from DC to 134 Hz. All signals were digitized with a sample rate of 512 Hz and 24-bit A/D conversion.

Offline, a mathematically linked average mastoid reference was applied and EEG and EOG activity was filtered with a bandpass of 0.10–30 Hz (phase shift-free Butterworth filters; 24dB/octave slope). Ocular correction was applied according to the Gratton, Coles and Donchin [29] algorithm. Artifact rejection criteria were minimum to maximum baseline-to-peak allowed voltage -100 to +100 μV, and a maximum allowed voltage skip (gradient) of 75 μV per sample point. The data were segmented in epochs of 900 ms, from 100 ms pre-target onset, serving as baseline, to 800 ms post-target onset. The mean 100 ms pre-stimulus period was used for baseline correction. The average number of trials included in the ERP analyses for the adults for Fair Accept was 44 (range = 30–56), for Fair Reject 29 (range = 11–43), for Unfair Reject 45 (range = 26–62), and for Unfair Accept 30 (range = 17–38). The average number of trials included in the ERP analyses for adolescents for Fair Accept was 44 (range = 26–63), for Fair Reject 29 (range = 12–40), for Unfair Reject 38 (range = 25–64), and for Unfair Accept 26 (range = 14–49).

The average number of trials for the alternatives was not high enough for a reliable signal, however we do report the average number and range of those trials for clarity reasons. In the fair-alternative condition (7/3 versus 5/5) the average number of accepted 7/3 trials for adolescents was 10 (range = 4–28), rejected trials 16 (range = 4–34). For adults the average number of 7/3 trials was for accepted 17 (range = 2–39) and for rejected 11 (range = 0–27). In the hyper-fair alternative condition (7/3 versus 3/7) the average number of accepted 3/7 trials for adolescents was 22 (range = 4–33), rejected trials 14 (range = 4–28). For adults the average number of 3/7 trials was for accepted 17 (range = 0–35) and for rejected 9 (range = 0–25).

## Results

### Behavioral results

**Fig 2:**
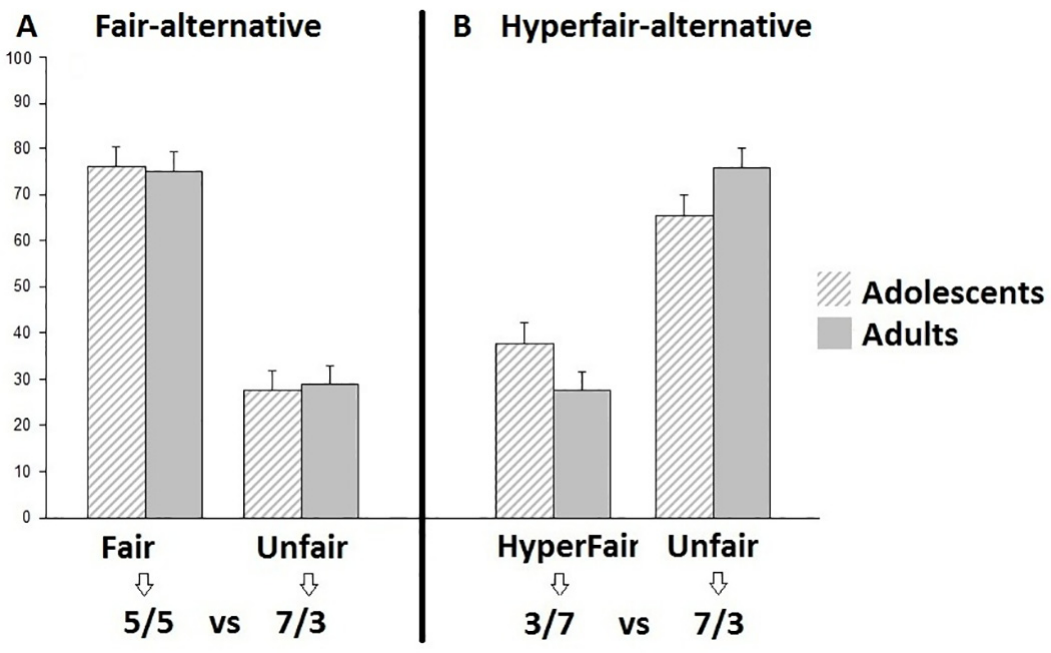
Behavioral results. In panel A the first column reflects the percentage of fair offers (5/5) and the second column reflects the percentage of unfair offers (7/3) made in the fair-alternative condition (5/5 vs 7/3). Both columns add up to 100%. In panel B the first column reflects the percentage of hyperfair offers (3/7) and the second column reflects the percentage of unfair offers (7/3) made in the hyperfair-alternative option (3/7 vs 7/3). Both columns add up to 100%. The error bars represent the standard error.

The percentage of unfair offers was examined in the fair-alternative condition in which an unfair distribution was pitted against a fair distribution (7/3 versus 5/5) and in the hyper-fair alternative condition where an unfair distribution was pitted against a hyperfair distribution (7/3 versus 3/7) (see Fig 2 for the percentages of the different offers made by the participant). The Condition (2) x Age group (2) repeated measures ANOVA for unfair choices resulted in a main effect of Condition (*F* (1,32) = 102.09, *p* < .001, ƞ^2^partial = .76), showing that participants made more unfair offers in the hyperfair-alternative (69%) than in the fair-alternative (27%) condition. There was neither a main effect of Age group (*F* (1,32) = 1.72, *p* = .20, ƞ^2^partial = .05), nor an interaction with Age group, (*F* (1,32) = 1.18, *p* = .29, ƞ^2^partial = .04) indicating that adolescents and adults made similar offers.

### ERP results

Grand averages were calculated for the fair offers (5/5) in the fair-alternative (5/5 vs 7/3) condition and unfair offers (7/3) in the hyperfair-alternative condition (3/7 vs 7/3). We focused on these two offers because the alternative offer types did not have enough trials for a reliable signal-to-noise ratio. Based on previous studies with the Ultimatum Game [16, 17, 20, 21, 23] and based on visual inspection, we defined the MFN component as the mean activity in the 240–320 ms time window after feedback presentation. To minimize potential effects of overlap of the MFN and other ERP components, such as the P300, we calculated difference waves (e.g. [16, 17, 20, 21, 30]). Both the amplitudes and the differences waves were analyzed and reported below.

We performed two repeated measures ANOVA to (1) test whether the MFN is modulated when a fair offer (5/5) is rejected compared to when it is accepted, reflecting a violation of fairness expectations and (2) to test whether the MFN responds to a violation of fairness-related expectations specifically or to unexpected events in general, by examining the MFN following unexpected acceptance.

**Fig 3:**
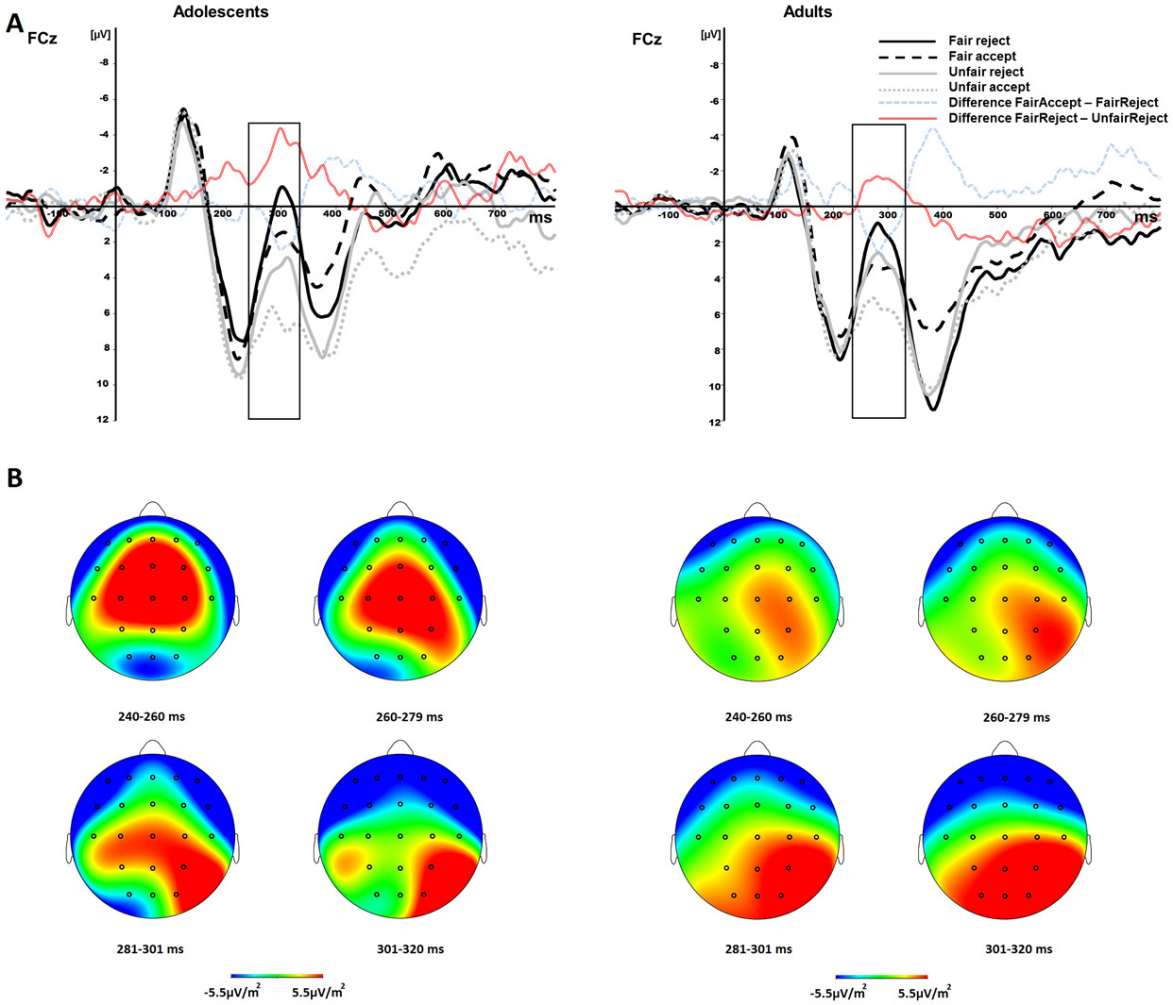
MFN amplitudes to acceptance or rejection of offers. MFN measured in the proposer after receiving feedback whether an offer is accepted or rejected. The horizontal axis shows time in milliseconds and vertical axis the electric potential in micro volt. Panel A reflects the MFN amplitudes (highlighted as the time-interval between 220–320 ms) for proposers after making fair and unfair offers. The MFN amplitudes for fair offers are plotted in black, while unfair offers are plotted in grey. The solid lines reflect MFN amplitudes after receiving feedback that an offer was rejected, while dashed lines reflect amplitudes after receiving feedback that an offer was accepted. Negative voltages are plotted upwards. In panel B the scalp potential topography is shown for the time point of the MFN (240–320 ms) after receiving feedback signaling whether an offer was accepted or rejected.

The first hypothesis we tested was whether the MFN would be more pronounced when a fair offer (5/5) was rejected compared to accepted. Therefore, we performed a 2 (Electrode: FCz, Cz) x 2 (Feedback: Accept vs. Reject) x 2 (Age group: Adolescents vs. Adults) repeated measures ANOVA for the MFN amplitude of fair offers (5/5) in the fair-alternative condition (5/5 vs 7/3). This analysis resulted in a main effect of Feedback, *F* (1,32) = 6.12, *p* = .019 and Electrode, *F* (1,32) = 48.49, *p* < .001. As can be seen in Fig 3, the MFN was larger for reject than for accept trials in both adolescents and adults. The MFN was also negative at FCZ, but there was no Feedback x Electrode interaction.

Next, we computed a difference wave by subtracting Fair Reject from Fair Accept. To test whether this difference of Fair Accept—Fair Reject significantly differed from zero we performed a one-sampled t-test on the difference wave of Fair Accept—Fair Reject (see Fig 3) for both FCz and Cz. The one-sample t-tests showed that the MFN as measured by the difference between Fair Accept—Fair Reject significantly differed from zero at FCz, *t* (33) = 2.46, *p* = .020 and at Cz, *t* (33) = 2.53, *p* = .016. Although the repeated measures ANOVA showed no interaction with Age group we performed an independent sample t-test with Age group as a between subjects factor to confirm there were no differences between adults and adolescents. Indeed, we did not find significant differences between adults and adolescents neither for FCz, *t* (32) = .18, *p* = .855, nor for Cz, *t* (32) = .11, *p* = .914.

To test the second hypothesis examining whether the MFN responded to violation of fairness-related expectations specifically or to unexpected events in general, we focused on unexpected acceptance of unfair offers. That is, when the MFN would be larger for acceptance than for rejection of unfair offers in the hyperfair-alternative condition this would indicate a general expectancy violation. Therefore, we performed a similar 2 (Electrode: FCz, Cz) x 2 (Feedback: Accept vs. Reject) x 2 (Age group: Adolescents vs. Adults) repeated measures ANOVA for the MFN amplitude of unfair offers (7/3) in the hyperfair-alternative condition (3/7 vs 7/3). The results showed a main effect of Feedback, *F* (1,32) = 7.63, *p* = .009, and Electrode, *F* (1,32) = 61.24, *p* < .001. The MFN was larger for reject than for accept trials in both adolescents and adults. The MFN was also negative at FCz, but again, there was no Feedback x Electrode interaction.

Next, we performed one-sample t-tests for the difference waves of Unfair Accept—Unfair Reject and found that the difference wave significantly differed from zero in FCz, *t* (33) = 2.88, *p* = .007 and in Cz, *t* (33) = 2.58, *p* = .014. Again, when we performed an independent sample t-test with Age group as a between subjects factor we found no significant differences between adults and adolescents for neither FCz, *t* (32) = .02, *p* = .984, nor for Cz, *t* (32) = .01, *p* = .990.

When directly comparing the rejection trials after a fair offer (5/5) in the fair-alternative condition (5/5 vs. 7/3) and after an unfair offer (7/3) in the hyperfair-alternative condition (3/7 vs. 7/3), the 2 (Electrode: FCz, Cz) x 2 (Feedback: Reject after fair offer vs. Reject after unfair offer) x 2 (Age group: Adolescents vs. Adults) repeated measures ANOVA revealed a main effect of Feedback, *F* (1,32) = 18.29, *p* < .001 and Electrode, *F* (1,32) = 65.48, *p* < .001. The MFN was larger (more negative; see also Fig 3) when a fair offer was rejected compared to when an unfair offer was rejected.

Finally, we performed a one-sample t-tests for the difference between Fair Reject—Unfair Reject showing that the difference wave significantly differed from zero in FCz, *t* (33) = 3.58, *p* = .001 and Cz, *t* (33) = 4.45, *p* = .001. However, there was no difference between the two age groups for neither FCz, *t* (32) = 1.36, *p* = .183, nor Cz, *t* (32) = 0.81, *p* = .426.

Thus for all analyses, there was no interaction between Feedback (accept vs. reject) and Age group, nor were there significant differences in the difference waves, indicating that in the current study adolescents and adults did not differ in manifestation of the MFN during rejection of fair offers in the fair-alternative and rejection of unfair offers in the hyperfair-alternative conditions.

## Discussion

The goal of this study was to test whether being rejected in a repeated bargaining game would result in the same neural response as what previously has been found for receiving an unfair offer when bargaining. In a modified Ultimatum Game, we were specifically interested in the neural responses to the acceptance or rejection of fair offers (5/5) made in the fair-alternative condition (5/5 vs. 7/3) and the acceptance or rejection of unfair offers (7/3) made in the hyperfair-alternative condition (3/7 vs 7/3). The results confirmed our expectation that being rejected after proposing a fair split of money (5/5) is associated with a larger MFN compared to being rejected after proposing an unfair distribution (7/3). The MFN was less pronounced when unfair offers were unexpectedly accepted, suggesting that this neural response is an index of a social prediction error, or norm violation of fairness expectancy (see [31] for a review). This effect was observed in adolescents and adults, suggesting that individuals between 15 and 21 years of age may be similarly sensitive to bargaining-related social rejection.

The results of this study suggest that the neural response to being rejected after offering a fair split of money as a proposer is comparable to the neural response of receiving an unfair offer as a responder [16, 17]. Although we did not compare the rejection of a fair offer directly to receiving an unfair offer, the pattern of the MFN does show a resemblance, such that the MFN is more pronounced after a rejection of a fair offer compared to acceptance of a fair offer and more pronounced after receiving an unfair offer compared to receiving a fair offer. Previously, this neural response was interpreted as a signal that detects deviance from social norms in ultimatum game paradigms [14, 16, 27]. The anterior cingulate cortex (ACC), a brain region which has been found to be the most likely source of the MFN [32], has previously been implicated in social rejection and social norm violation [14]. For example, using neuroimaging, it was previously found that social exclusion results in activation in the ACC and the insula, regions which have been associated with experiencing physical as well as social pain [33, 34] (see [35] for a review). In addition, rejecting a fair offer in an Ultimatum Game when someone usually accepts these offers also results in activation in the ACC and insula [25]. Also, deciding to divide a sum of money unequally results in activation in the dorsal ACC and insula [36]. Possibly the MFN reflects a negative prediction error, which signals the dismissal when someone deviates from social norms [16, 17, 20, 21, 22, 23]. In several studies it was found that the MFN was modulated by the degree of unfairness of an offer [16, 22, 24]. The MFN is more pronounced when participants receive a highly unfair offer compared to moderately unfair offers. Individual differences in concern for fairness affect the amplitude of the MFN [17]. In the current study the rejection of a fair offer (5/5) may be experienced as more unfair since the responder was aware of the alternative, namely an unfair offer (7/3). Previous research has shown that proposers’ intentionality affects rejection rates of responders when a proposer deviates from the social norm, such that responders reject unfair offers more when paired with a fair alternative [37].

Interestingly, the rejection sensitivity was larger when being rejected after offering a fair split compared to being accepted offering an unfair split, although both may be interpreted as an unexpected event. It has repeatedly been found that unfair offers are often rejected (e.g. [2, 10, 12]) no matter the eventual cost for the responder. Therefore, it is likely that the proposer expected responders to accept fair offers and reject unfair offers. Possibly, the higher MFN after being rejected following a fair offer can be interpreted as an index of social pain that is experienced following rejection, over and above the notion that this is unexpected. Alternatively, the MFN may reflect general (non-social) prediction errors, since the predicted value of a fair offer is always higher than that of an unfair (or hyperfair) offer (e.g. [18]). Future studies should test these hypotheses in more detail, for example, by including a broader range of affective measures such as self-report and autonomic reactions (e.g. [38]).

One of the goals of this study was to examine if the MFN response to violation of the fairness expectancies of the proposer was larger in adolescents than in adults. Prior research has been inconclusive with respect to whether social rejection sensitivity is more salient in mid- to late adolescence than early adults, some supporting this notion based on self-reports on rejection sensitivity [39]. However, in a peer rejection game where participants received rejection feedback based on first impressions, it was found that all groups showed similar activation in medial prefrontal cortex (mPFC) when being accepted [40]. In addition, other studies examining social exclusion reported similar levels of activation in anterior medial frontal cortex (amPFC) and insula in adolescents and adults [41, 42, 43, 44]. Consistent with these prior studies, no differences were found between 15-year-old adolescents and 20-year-old young adults in MFN activity after bargaining rejection, suggesting that the signals that originate from the ACC after violation of expectancies or predictions related to responses to fairness and unfairness were similarly present in adolescence. These findings fit with prior developmental results showing the same neural response in the ACC and insula in children, adolescents and adults to diversions from normative behavior [41]. The current results are also consistent with a prior psychophysiological study showing that heart rate responses following peer rejection originated in puberty and was at adult levels by mid-adolescence [38].

Only few studies have examined the Feedback Related Negativity (FRN; comparable to MFN) directly comparing mid-adolescents with adults [45, 46,47]. Results from these studies have been inconclusive as to whether mid-adolescents already show adult-like FRN responses, which would suggest more efficient feedback processing [48]. For instance, Santesso et al. [46] found adult-like FRN responses for 16–17 year olds compared to 18–29 year olds. However, Hämmerer et al. [45] found a linear increase with age in FRN amplitude in 13-14-year-olds versus adults, where Zottoli and Grose-Fifer [47] found that 14–17 year olds showed a larger FRN amplitude but differentiated less between loss and gain feedback compared to 22–26 year olds. In light of these results and the relatively low sample size of the current study it remains a question for future research whether the similar MFN results for our mid-adolescent and adult group reflects a similar proficiency in processing feedback and or/signaling of norm violation. Future studies should employ bigger sample sizes and include a wider age range to examine the development of MFN/FRN in a social norm violation paradigm (see [49] for a review). Also, examining a wider range of components in the whole epoch, such as the P300, with more advanced analyses methods, including mass univariate analyses [50] can provide a clearer picture on the underlying mechanisms of fairness considerations (see [51] and [52] for a review).

### Limitations

In the current study we chose to have participants play continuously with the same player during the entire experiment to buildup social expectations [17, 21]. However, such an experimental setup may alter expectancies as the session progresses. Previous research has shown that the FRN/MFN changes with expectancy when participants behaviorally learn (e.g. [18, 53, 54, 55, 56]). However, there also have been reports in which the FRN remained constant as response accuracy increased, or even that participants learn without any clear FRN [45, 57, 58], (see [31] for a review). Future studies should take into account that the buildup of expectancies may alter the FRN during the UG when playing with the same player. Furthermore, it may be possible that the color of the given feedback may have inflated the MFN, such that the negative reject feedback in red elicited an even larger MFN compared to when the negative reject feedback would have been given in a more neutral color. However, even if the color of the feedback inflated the MFN this would not have affected the critical comparison of negative reject feedback after a fair proposal and negative reject feedback after an unfair proposal, because in both conditions the feedback had a red color.

### Conclusion

Taken together, the current study was the first to show that the MFN, a neural signal originating from the ACC, is sensitive to rejection and social prediction errors of fairness considerations in bargaining situations. Whereas prior studies focused on the modulation of the MFN in responders who receive unfair offers, the current study examined the neural response in the proposer and showed that the MFN is also present when fair offers of proposers are rejected. Prior studies revealed that the MFN response to norm violations is dependent on social context. For example, it was previously found that the error-related negativity (ERN), which is observed when people make errors, is larger for people with low social status [59]. In addition, the MFN is more negative going when receiving an unfair offer from a stranger (when status still needs to be determined) than when receiving an unfair offer from a friend ([21], but see [23]). An important direction for future research is to examine the determinants of rejection sensitivity of proposers, for example by manipulating the status of the proposer and responder. These results are expected to provide important information about how individuals make choices for fairness and reciprocity, which are important processes for developing social ties.

## Supporting Information

S1 Appendix Cover story. (DOCX)
