# Supplementary material for: Rejection in Bargaining Situations: An Event-Related Potential Study in Adolescents and Adults
Source: PLoS One. 2015 Oct 7;10(10):e0139953. doi: 10.1371/journal.pone.0139953 (PMC4596717; doi:10.1371/journal.pone.0139953)
Supplement: S1 Appendix — (DOCX) [file pone.0139953.s001.docx]

**S1 Appendix. Cover story**

Upon arrival in the lab the experiment leader and participant read through an informed consent letter in which, amongst others, the purpose of the study was discussed. The participant was explained that the study focused on social interactions and therefore the participant would be playing with another player. The participant was led to believe that the other player was located in a lab within the same building, but that they could not meet due to anonymity reasons. In the letter of consent the importance of anonymity was also highlighted (stating that all data would be anonymized). The participant was instructed that the experiment leaders would keep in contact by telephone or by visiting the other player’s lab. The participant was also informed that the other player was of the same gender and approximately the same age.

During preparations of connecting the EEG equipment a confederate would walk into the room introducing him- or herself to the participant as the experiment leader of the other player. After introductions were made the confederate asked how the preparations were proceeding and asked an estimation on how long it would take before the experiment could start. In return, the experiment leader asked how preparations in the other lab were going. They agreed to keep each other posted by telephone. By the end of the preparations and during breaks the telephone would ring and the participant would hear the experiment leader talking to the confederate about when to start (or resume) the experiment.

Afterwards, all participants were asked how they experienced the study to test if they believed the cover story. Then, all participants were debriefed.
